# Calpain inhibition rescues troponin T3 fragmentation, increases Cav1.1*,* and enhances skeletal muscle force in aging sedentary mice

**DOI:** 10.1111/acel.12453

**Published:** 2016-02-19

**Authors:** Tan Zhang, Andrea S. Pereyra, Zhong‐Min Wang, Alexander Birbrair, Julie A. Reisz, Daniel Clark Files, Lina Purcell, Xin Feng, Maria L. Messi, Hanzhong Feng, Joseph Chalovich, Jian‐Ping Jin, Cristina Furdui, Osvaldo Delbono

**Affiliations:** ^1^ Department of Internal MedicineSection on Gerontology and Geriatric MedicineWake Forest School of MedicineWinston‐SalemNCUSA; ^2^ Molecular Medicine and Translational ScienceWake Forest School of MedicineWinston‐SalemNCUSA; ^3^ Pulmonary, Critical Care, Allergy and Immunologic DiseasesWake Forest School of MedicineWinston‐SalemNCUSA; ^4^ Department of OtolaryngologyWake Forest School of MedicineWinston‐SalemNCUSA; ^5^ Wayne State University School of MedicineDetroitMIUSA; ^6^ Department of Biochemistry and Molecular BiologyBrody School of MedicineEast Carolina UniversityGreenvilleNCUSA; ^7^ J Paul Sticht Center on AgingWake Forest School of MedicineWinston‐SalemNCUSA; ^8^ Present address: Biochemistry Research Institute of La Plata (INIBIOLP)/CONICETSchool of MedicineNational University of La Plata1900La PlataBAArgentina; ^9^ Present address: Ruth L. and David S. Gottesman Institute for Stem Cell and Regenerative Medicine, Albert Einstein College of MedicineNY10461USA; ^10^ Present address: Department of Biochemistry and Molecular GeneticsUniversity of Colorado DenverAuroraCO80045New YorkUSA

**Keywords:** aging, calcium channel, calpain, excitation–contraction coupling, skeletal muscle, troponin T

## Abstract

Loss of strength in human and animal models of aging can be partially attributed to a well‐recognized decrease in muscle mass; however, starting at middle‐age, the normalized force (force/muscle cross‐sectional area) in the knee extensors and single muscle fibers declines in a curvilinear manner. Strength is lost faster than muscle mass and is a more consistent risk factor for disability and death. Reduced expression of the voltage sensor Ca^2+^ channel α1 subunit (Cav1.1) with aging leads to excitation–contraction uncoupling, which accounts for a significant fraction of the decrease in skeletal muscle function. We recently reported that in addition to its classical cytoplasmic location, fast skeletal muscle troponin T3 (TnT3) is fragmented in aging mice, and both full‐length TnT3 (FL‐TnT3) and its carboxyl‐terminal (CT‐TnT3) fragment shuttle to the nucleus. Here, we demonstrate that it regulates transcription of *Cacna1s*, the gene encoding Cav1.1. Knocking down TnT3 *in vivo* downregulated Cav1.1. TnT3 downregulation or overexpression decreased or increased, respectively, *Cacna1s* promoter activity*,* and the effect was ablated by truncating the TnT3 nuclear localization sequence. Further, we mapped the *Cacna1s* promoter region and established the consensus sequence for TnT3 binding to *Cacna1s* promoter. Systemic administration of BDA‐410, a specific calpain inhibitor, prevented TnT3 fragmentation, and *Cacna1s* and Cav1.1 downregulation and improved muscle force generation in sedentary old mice.

## Introduction

Aging is associated with loss of muscle strength and power that contributes to fall risk, impaired mobility, and reduced quality of life (Alley *et al*., 2010). Besides its recognized role in mobility, posture, heat regulation, and endocrine function, the skeletal muscle plays a central role in whole body metabolism, which affects systemic aging and lifespan (Demontis *et al*., 2013). Cohort studies show that strength is lost two‐ to five‐times faster than muscle mass with age (Goodpaster *et al*., 2006), and its loss is a more consistent risk factor for disability and death (Newman *et al*., 2006). Although muscle mass is one determinant of strength, its loss does not fully account for aging‐related strength loss (Rolland *et al*., 2007). Therapies that increase not only muscle mass but muscle strength, power, and quality (strength per unit of mass) would greatly benefit the health of older adults.

Our laboratory and others reported that aging impairs muscle activation–contraction efficiency (Delbono, 2011). Altered transmittal of membrane depolarization to SR Ca^2+^ release decreases force‐generation capacity in old rodents and humans (Wang *et al*., 2000). The molecular mechanism responsible for the loss of muscle‐contraction efficiency is decreased voltage‐gated calcium channel α1 subunit (Cav1.1) (Renganathan *et al*., 1997). It is essential for muscle contraction and, with aging, uncouples more ryanodine receptors (RyR1s) through an undefined mechanism.

In the myoplasm, troponin T (TnT) is known to mediate the interaction between the Tn complex and tropomyosin (Tm), which is essential for Ca^2+^‐activated striated muscle contraction (Jin *et al*., 2008). We reported a noncanonical role for TnT3, the TnT isoform expressed in fast‐twitch muscle fibers. We found full‐length (FL)‐TnT3 and its fragments in both the nuclear and cytosolic fractions of myofibers isolated from mouse skeletal muscle. More important, the myonuclei of old mice had less of the full‐length protein and more of the COOH‐terminal (CT) fragment (TnCT) than those of young mice (Zhang *et al*., 2013a,b). Whether TnT3 regulates expression of Cav1.1 in muscle *in vivo* is unknown.

Calpains are a family of calcium‐dependent cysteine endopeptidases. The skeletal muscle contains ubiquitous calpain‐1 (μ‐type) and calpain‐2 (m‐type), and muscle‐specific calpain‐3. Calpain‐1 mediates proteolysis of various cellular proteins, including cytoskeletal proteins (Campbell & Davies, 2012). Calpain‐1 overactivation causes irreversible cell damage, contributing to the pathology of cerebral and cardiac ischemia, Alzheimer's disease, arthritis, and cataracts (Wang & Yuen, 1994; Lee *et al*., 2000). Calpains may be activated by muscle microinjuries throughout the lifespan, particularly at later stages, when increased susceptibility to trauma may activate calpains (Faulkner *et al*., 1993; Salazar *et al*., 2010).

In this study, we proved our hypothesis that TnT3 regulates Cav1.1 expression in fast adult myofibers. Decreased nuclear FL‐TnT3 and increased CT‐TnT3 fraction result in decreased expression of *Cacna1s*, the gene encoding Cav1.1, and impaired excitation–contraction coupling (ECC) with aging. They can be rescued in sedentary old mice by systemic administration of BDA‐410, a specific calpain inhibitor, which prevents TnT3 fragmentation.

## Results

### TnT3 regulates *Cacna1s* transcription and Cav1.1 expression

**Figure 1:**
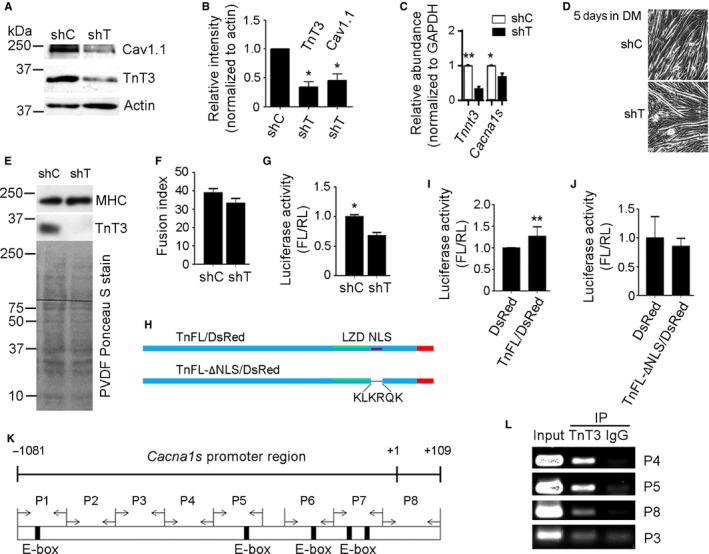
TnT3 knockdown *in vivo* decreases Cav1.1 and *Cacna1* expression; its overexpression enhances *Cacna1s* promoter activity in C2C12 and mouse muscle *in vivo*; and it is recruited onto the *Cacna1s* promoter. (A) Representative immunoblot of protein extracts from shC‐ and shT‐electroporated FDB muscles. (B) Quantification of 3 independent immunoblots, showing that shT decreases TnT3 and Cav1.1. (C) Quantitative qRT–PCR showing that shT successfully knocked down *Tnnt3* mRNA, which resulted in downregulation of *Cacna1s*. *P < 0.05, **P < 0.01. (D) Cultured C2C12 cells transfected with shC or shT did not show differences in myotube formation at day 5 day in DM. (E) Immunoblot of total protein extracts. Compared to shC‐, shT‐transfected C2C12 myotubes show strong TnT3, but not MHC, knockdown. PVDF membrane stained with Ponceau S indicates the same total protein loading. (F) Fusion index shows that shT treatment did not prevent C2C12 myoblast fusion at day 5 in DM. (G) Luciferase activity assay, expressed as the firefly/renilla ratio, shows that knocking down TnT3 inhibits *Cacna1s* promoter activity in C2C12 cells at day 5 in DM. (*P < 0.05, **P < 0.01). (H) Diagram of nuclear localization sequence (NLS) and leucine zipper domain (LZD) deletion in the full‐length TnT3 (TnFL)‐DsRed fusion construct. (I) Raw normalized data comparing luciferase activity for TnFL, TnFL‐ΔLZD/DsRed, TnFL‐ΔNLS/DsRed, and DsRed. Differences between these individual groups and DsRed are noted by their level of significance (*) *P* < 0.05 or (**) *P* < 0.005. (J) Primer set used to amplify mouse *Cacna1s* promoter regions that may interact with TnT3. Numbers indicate distance from the transcription start site. Eight primer pairs were designed to walk along regions P1‐P8. (K) Representative ChIP‐PCR experiment in C2C12 myotubes showing *Cacna1s* promoter regions recruiting endogenous TnT3. IgG was used as a control.

To test whether TnT3 regulates *Cacna1s* transcription, we knocked down TnT3 in mouse skeletal muscle *in vivo* to determine whether Cav1.1 expression depends on TnT3 regulation of *Cacna1s*. We compared flexor digitorum brevis (FDB) muscles 3 weeks after electroporation with control nontargeting (shC) or targeting TnT3 shRNA (shT) by immunoblot, and quantitative real‐time PCR (qPCR) (Fig. 1A–C). shT's efficiency in knocking down TnT3 was confirmed by immunoblot (Fig. 1A,B), which showed an over 70% (*P* < 0.05) decrease in endogenous TnT3 expression. Using this preparation, we found that Cav1.1 was reduced by ~ 60% (*P* < 0.05) due to downregulation of *Tnnt3* (*P* < 0.01) and *Cacna1s* (*P* < 0.05) mRNA (Fig. 1C).

**Figure 2:**
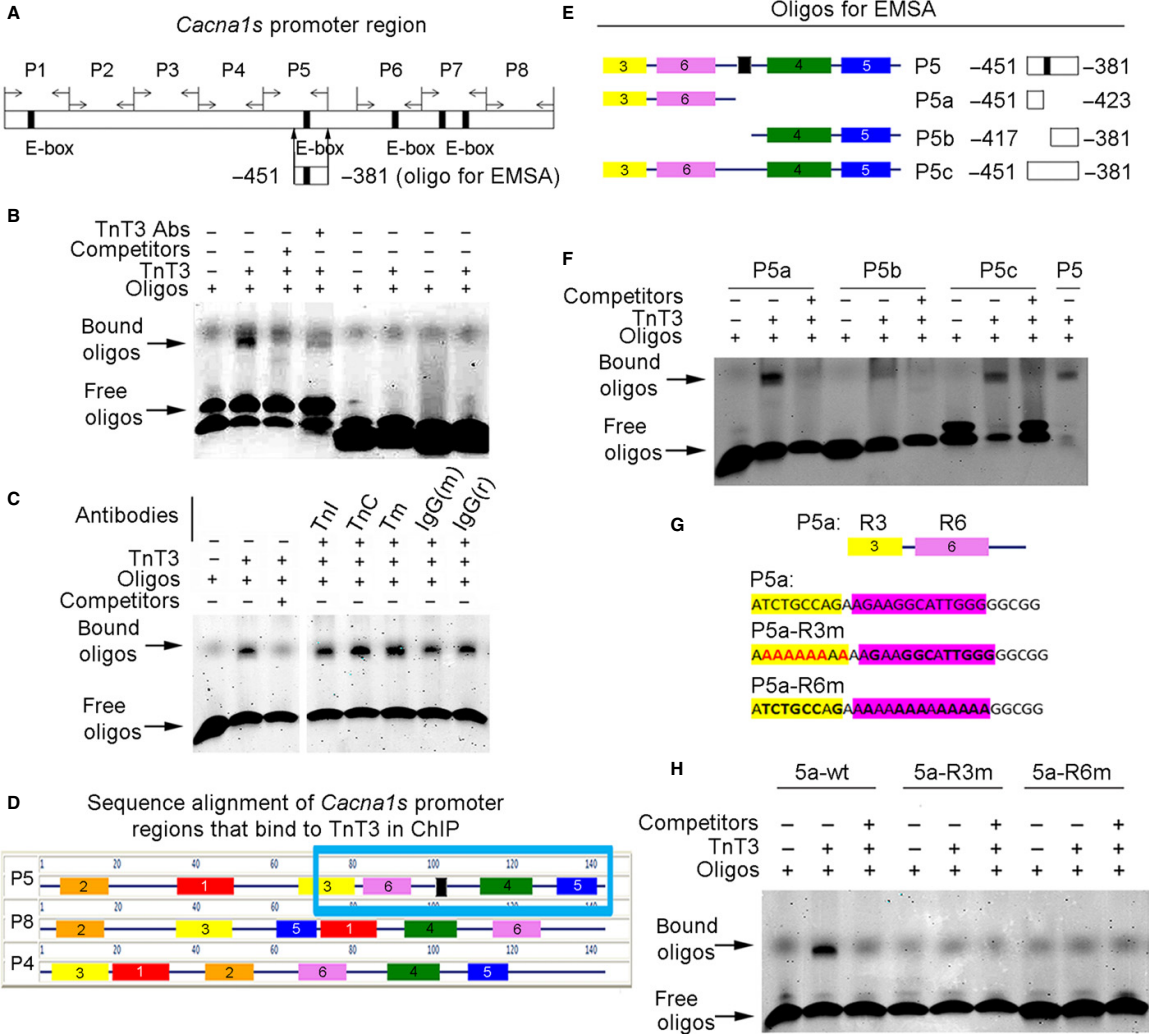
EMSA mapping of the *Cacna1s* promoter region that interacts with TnT3. (A) EMSA oligonucleotide designed to test the proximal half of the *Cacna1s* promoter's P5 region and used in subsequent experiments. (B) Compared to oligos alone (lane 1), TnT3 induces a band shift (lane 2) that is attenuated by unlabeled oligos (lane 3) and pre‐incubation with TnT3 and TnT3 antibody (lane 4). Two oligos, designated control‐ and control‐2 (Table S1), unrelated to *Cacna1s*, show no binding to TnT3 (lanes 4–8). (C) Compared to P5 oligos alone (lane 1), TnT3 induced a band shift (lane 2) that was attenuated by unlabeled oligos (lane 3). In contrast, TnI, TnC, and Tm antibodies and mouse (m) and rabbit (r) IgG did not attenuate band shift as shown in a separate gel. (D) Multiple EM for Motif Elicitation (MEME) (Bailey et al., 2006) sequence alignment and comparison of P4, P5, and P8 regions. The six consensus motifs identified are highlighted. The blue box encloses the *Cacna1s* promoter region that interacts with TnT3 in the ChiP assay. (E) Oligonucleotide design, based on MEME, identified motifs in the P5 proximal half region. P5a is the sequence upstream the E‐box (in black); P5b is the sequence downstream of the E‐box; and P5c is the E‐box in the mutated full‐length P5. (F) Representative EMSA data show that P5b has the weakest binding to TnT3, while P5a shows strong binding. The E‐box does not seem required for TnT3 binding because both P5c (E‐box mutant) and P5a (sequence upstream of E‐box) clearly bind to TnT3. (G) Diagrams showing oligos with mutated P5a‐R3 (P5a‐R3 m) or P5a‐R6 (P5a‐R6 m). H) Representative EMSA data showing that both R3 and R6 mutations diminished the gel shift of P5a oligos.

To examine the hypothesis that TnT3 regulates *Cacna1s* transcription, we performed a dual luciferase assay using a construct in which the *Cacna1s* promoter drives the firefly luciferase reporter gene (Zheng *et al*., 2002a). We analyzed C2C12 myotubes transfected with shC or shT at day 5 in differentiation medium (DM) (Fig. 1D), when *Cacna1s* promoter activity peaks (Zheng *et al*., 2002a). The shT effectively knocked down TnT3 but not myosin heavy chain (MHC) protein levels (Fig. 1E); accordingly, *Cacna1s* promoter activity was inhibited (Fig. 1G), while myotube formation and differentiation capacity, reflected by the fusion index and MHC level, respectively, was not altered significantly (Fig. 2E,F). Compared to control DsRed, TnFL‐DsRed but not the nuclear localization signal (NLS)‐deletion construct TnFL‐ΔNLS/DsRed or the leucine zipper domain (LZD)‐deletion construct enhanced *Cacna1s* promoter activity in mouse FDB muscle *in vivo* (Fig. 2H–I). These results indicate that (i) TnT3 enhances *Cacna1s* promoter activity, (ii) TnT3 knockdown directly reduces *Cacna1s* promoter activity in skeletal muscle, and (iii) preventing TnT3's nuclear translocation inhibits its effect on *Cacna1s* transcription.

### TnT3 interacts with the *Cacna1s* promoter region

Next, we examined TnT3 recruitment onto the *Cacna1s* promoter using a ChIP‐based promoter walkthrough analysis. We tested eight pairs of PCR primers, covering the full‐length of the *Cacna1s* promoter region (−1081 to +109), and found three regions (P4, P5, and P8) that may recruit TnT3 (Fig. 1J,K). As P5 contains an E‐box motif, which is known to interact with a leucine zipper domain (Vinson *et al*., 1989) that is found in TnT3 (Zhang *et al*., 2013a), we explored whether TnT3 directly binds this region. We used an electrophoretic mobility shift assay (EMSA) and the IRDye700‐labeled double‐stranded oligonucleotide (*Cacna1s* −451 to −381) containing an E‐box (Fig. 2A). When incubated with TnT3 purified from mouse tibialis anterior muscle, the IRDye700‐labeled wild‐type 170‐bp probe exhibited gel shift, which was inhibited by adding 200‐fold molar excess of unlabeled oligonucleotides. The shift was consistently attenuated by adding a TnT3‐specific antibody during incubation. In contrast, two other oligonucleotides containing sequences other than the *Cacna1s* promoter's showed no gel shift in the presence of TnT3 (lanes 5–8) (Fig. 2B).

To rule out any contribution from TnI, TnC, and/or Tm contaminating the EMSA signal, we performed these experiments with their respective antibodies. In contrast to the TnT3 Ab, they did not attenuate the TnT3/P5 oligonucleotide interaction (Fig. 2B,C). These results demonstrate the specificity of TnT3 binding to the *Cacna1s* promoter region (−451 to −381) and its independence from Tn‐Tm complex formation.

Establishing that TnT3 is recruited to the *Cacna1s* P4, P5, and P8 promoter regions by ChIP‐PCR (Fig. 1L), we next used sequence alignment to explore their conserved consensus binding motifs. From the six identified, three complete consensus motifs (Fig. S1) were found in the P5 probe (−451 to −381) and P4 and P8 regions (blue box in Fig. 2D). EMSA analysis of a further truncated P5 probe showed that TnT3 binds to the P5a region (−451 to −423) (Fig. 2E,F). E‐box mutation (P5c) or deletion (P5a) did not affect TnT3 binding, and P5b (−417 to −381) did not seem to play a major role in TnT3 binding (Fig. 2E,F). These data map the TnT3‐specific binding sequence to the −451 to −423 *Cacna1s* promoter region, which contains consensus motif 6 and part of 3. Mutation of either motif inhibited TnT3‐induced gel shift (Fig. 2G,H), indicating that TnT3 binds to each independently. TnT3 binding to the *Cacna1s* promoter region is consistent with our previous report that most transcription factor binding sites are located within 1 kb upstream of the *Cacna1s'* transcription start site (Zheng *et al*., 2002b).

### The TnT3 endogenous cleavage site in muscle *in vivo* is compatible with calpain activity

Figure S2A shows SDS‐PAGE separation of nuclear protein extracted from old mice in two lanes, one stained on gel with Coomassie blue, while the other was transferred to a PVDF membrane and immunoblotted. This TnT3 fragment corresponds to CT‐TnT3 (red box in gel and yellow box in immunoblot) as confirmed by mass spectrometry sequence analysis. The relative position of the CT‐TnT3 fragment in nuclear extracts was also determined with reference to the protein ladder. Figure S2B shows mass spectrometry analysis of trimethoxyphenyl phosphonium (TMPP)‐labeled nuclear protein extracts. TMPP‐labeled TnT3 N‐terminal amino acids are shown in red. GPS‐CCD 1.0 (Liu et al., 2011) indicates that two of three of the TMPP‐labeled sites are calpain cleavage sites.

### BDA‐410 inhibits endogenous skeletal muscle calpain activity

As calpain may be the enzyme that endogenously cleaves TnT3, we asked whether BDA‐410, a potent and selective inhibitor of calpain protease activity, would significantly reduce skeletal muscle calpain activity. Figure S3A shows that BDA‐410 significantly reduces AMC (7‐amino‐4‐methylcoumarin) cleavage. In mouse muscles treated with either vehicle (B) or BDA‐410 (C), enzyme activity was significantly decreased in the presence of calpain inhibitor III (B). Note that the BDA‐410‐treated muscles showed less enzyme activity than vehicle‐treated muscles in the absence of inhibitor (C), while in its presence, AMC activity was similar (B, C). These results indicate that BDA‐410 potently blocks endogenous calpain activity.

### Calpain inhibition enhances muscle force, but not fatigue, in old mice

**Figure 3:**
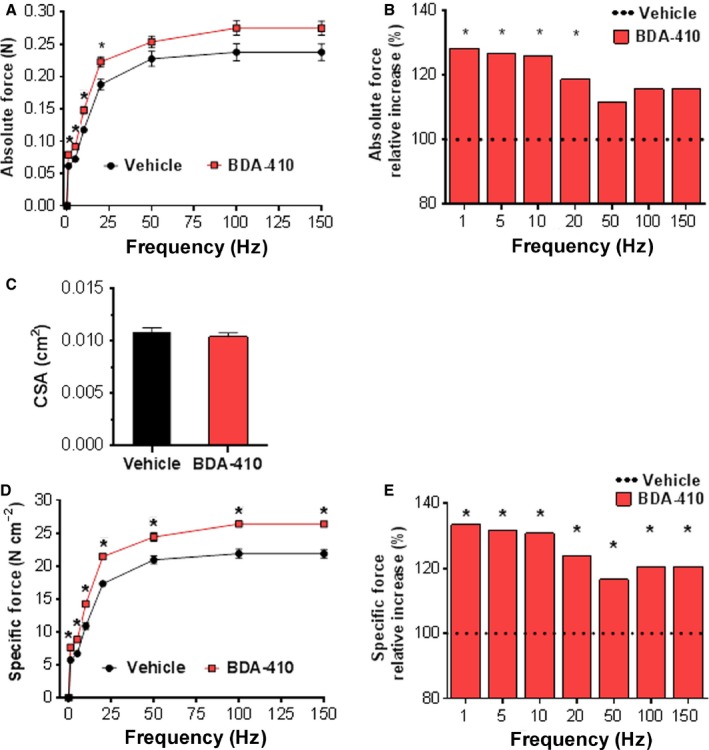
BDA‐410 increases *ex vivo* isometric contraction force in the soleus muscle. (A) Absolute force production in response to supramaximal stimulation at 1, 5, 10, 20, 50, 100 and 150 Hz. (B) Relative increase in absolute force in BDA‐410 compared to vehicle‐treated mice. (C) Soleus muscle cross‐sectional area (CSA). (D) Soleus muscle‐specific force calculated as the absolute force normalized to muscle CSA. (E) Relative increase in specific force in BDA‐410 compared to vehicle‐treated mice. A and B: *P < 0.01. D and E: *P ≤ 0.003. Left and right soleus muscles were included in the analysis. Data values are expressed as mean ± SEM. *n* = 6 for each group.

For these experiments, we chose the soleus muscle, which, unlike fast hindlimb muscles, is a mixture of fast and slow fibers and, in correlation with histology, can clarify any functional impact of changes in fiber‐type composition. Soleus muscle absolute force was significantly enhanced at submaximal and maximal stimulation rates in mice treated with BDA‐410 rather than vehicle (Fig. 3A,B). This stimulatory effect was not associated with increased muscle cross‐sectional area (CSA) (C), which indicates that the increase in muscle absolute force was due to increased efficiency, not protein accrual. The significant increase in specific force at the same stimulation frequency range in mice treated with BDA‐410 rather than vehicle further supports this conclusion (D, E).

Analysis of the contractile properties of the soleus and triceps surae muscles, using comparable protocols, showed no difference in the fatigue index either *ex vivo* or *in vivo* (Fig. S4A,B). We confirmed that BDA‐410 had no effect on muscle endurance by measuring the maximal tolerated speed in a forced treadmill (C). In contrast, inverted‐cling grip holding time, a measurement of strength, was significantly increased in BDA‐410 compared to vehicle‐treated mice (D).

### Calpain inhibition does not modify the overall levels of main motor and regulatory proteins or fiber type in fast and slow muscles from old mice

**Figure 4:**
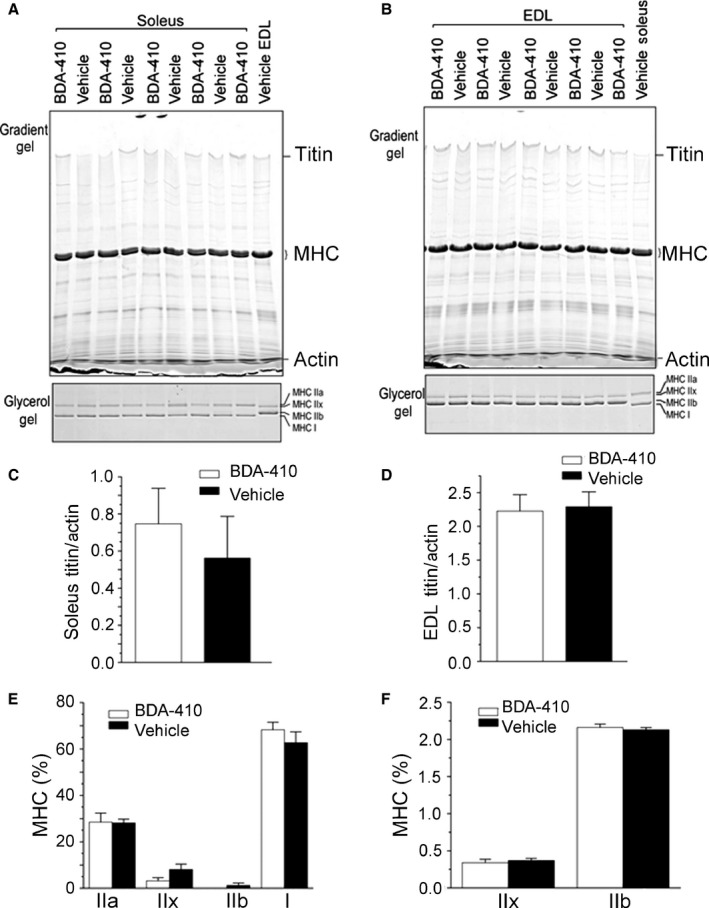
Calpain inhibition does not modify EDL and soleus fiber‐type composition or titin protein levels. Titin and MHC isoforms (IIa, IIx, IIb, and I) in soleus (A) and EDL (B) muscles measured in 5 mice treated with BDA‐410 and 4 treated with vehicle. Differences were not statistically significant (C–F).

Figure 4 shows that BDA‐410 does not modify EDL and soleus fiber‐type composition (IIa, IIx, IIb, and I) (A, B, E, F) or titin protein levels (C, D). Figure S5 shows that calpain inhibition does not modify the levels of myosin heavy chain (MHC) and actin or their ratio in either soleus (A) or EDL (B) muscles from BDA‐410‐ and vehicle‐treated mice (C). Figure S6 shows that calpain inhibition does not modify total fast or slow troponin T or troponin I. Total fast and slow TnT and TnI protein levels measured by immunoblot in soleus (A, B, E) and EDL (C‐D, F) muscles. Overall, these results indicate that increased muscle force in response to calpain inhibition cannot explained by main motor and regulatory protein accrual or switch in fiber‐type composition.

#### BDA‐410 stabilizes nuclear TnT3 integrity in skeletal muscle from old mice *in vivo*

**Figure 5:**
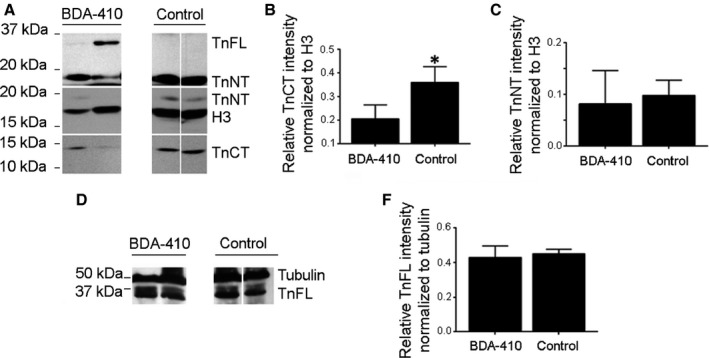
BDA‐410 stabilizes nuclear TnT3 integrity in old mice skeletal muscle *in vivo*. (A) Immunoblot of nuclear protein extractions from old mice treated with BDA‐410 or vehicle. CT‐TnT3 and TnNT were detected with antibodies targeting the C‐ or N‐terminal regions of TnT3. Histone H3 antibody was used to detect H3 as an internal nuclear protein loading control. (B) BDA‐410 effectively reduced the abundance of nuclear CT‐TnT3 (*P < 0.05), but not TnNT normalized to H3 (C) or TnFL normalized to tubulin (D–E).

In contrast to old mice treated with vehicle only, those treated with BDA‐410 had significantly lower levels of TnCT (Fig. 5A,B), while the TnNT fraction normalized to the nuclear protein marker histone H3 protein or TnFL normalized to tubulin (D‐E) did not differ significantly (C). Lack of change in total TnT protein levels (Fig. S6), despite decreased TnT3 fragmentation induced by BDA‐410 (Fig. 5A,B), is explained by the abundance of myofilament‐attached TnFL (Zhang *et al*., 2013a) and its dominance in immunoblot assays.

#### Calpain inhibition rescues Cav1.1 protein levels in old mice

**Figure 6:**
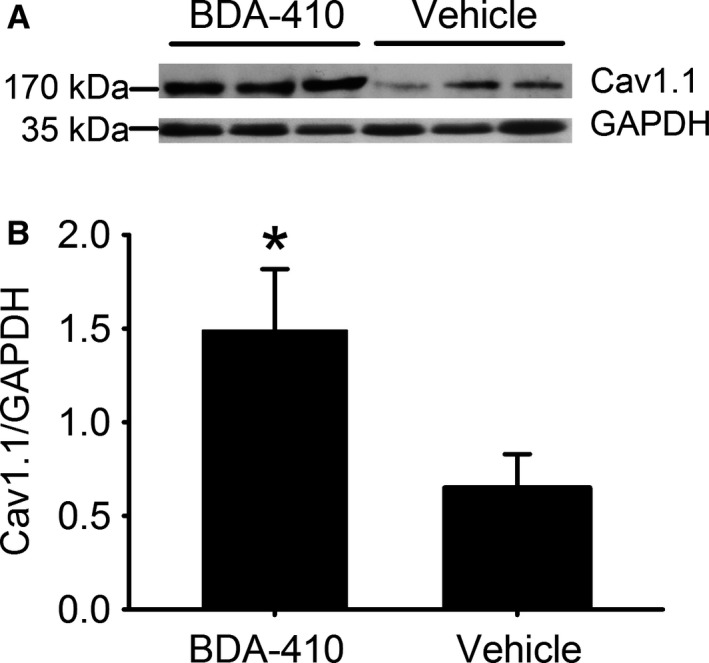
Cav1.1 protein levels are higher in old mice treated with BDA‐410 than with vehicle. (A) Cav1.1 and GAPDH immunoblot in pooled hindlimb muscles from old mice treated with either BDA‐410 (*n* = 3) or vehicle (*n* = 3). (B) Cav1.1 expression, normalized to GAPDH, analyzed by densitometry, is significantly higher in the old mice treated with BDA‐410 (*P < 0.05).

Figure 6A shows that Cav1.1 expression normalized to GAPDH is significantly higher in old mice treated with BDA‐410 rather than vehicle (*P* < 0.05). Thus, TnT3 fragmentation impairs *Cacna1s* transcription and Cav1.1 expression (Fig. 1), and BDA‐410‐mediated calpain inhibition rescues Cav1.1 levels in skeletal muscle from aging sedentary mice.

## Discussion

Here, we report three novel findings: (i) TnT3 regulates *Cacna1s* transcription and Cav1.1 expression in skeletal muscle fibers; (ii) calpain‐mediated decreased nuclear FL‐TnT3, and increased CT‐TnT3 fragment leads to decreased *Cacna1s* expression and impaired ECC; and (iii) systemic administration of BDA‐410 prevents TnT3 fragmentation and Cav1.1 downregulation in sedentary old mice.

Tropomyosin (TM)‐binding troponin (TnT) together with the calcium‐binding troponin C (TnC) and the inhibitory subunit troponin I (TnI) form a complex that regulates muscle contraction. Specifically, it interacts with actin and binds calcium to trigger production of muscle force (Gordon *et al*., 2000). In addition to this classical cytoplasmic location and function, the fast skeletal muscle TnT3 shuttles to the nucleus (Zhang *et al*., 2013a), and because it has a DNA‐binding domain (Zhang *et al*., 2013a), we hypothesized that TnT3 plays a nonclassical role in gene transcription. Recent publications show that TnI, TnT, Tm, and other cytoskeletal proteins shuttle to the nucleus in various cells. As TnT3, TnT1, and TnT2 exhibit a classical LZD (Vinson *et al*., 1989), TnT1 and TnT2 may have a nuclear function similar to that described for TnT3 in the present study. TnI has been reported in the nucleus of Drosophila nonmuscle cells (Sahota *et al*., 2009) and TnI and TnT in rat and human cardiomyocytes (Asumda & Chase, 2012); however, their function in these locations is largely unknown. We consistently demonstrated that the nonmyofilament‐associated fast skeletal muscle TnT3 enters the nucleus through a COOH‐terminus nuclear localization sequence (NLS), KLKRQK (Zhang *et al*., 2013a). Here, we demonstrate that TnT3 regulates gene transcription, playing a heretofore unknown role in nuclear signaling. We mapped the *Cacna1s* promoter region and established that TnT3 is recruited to and strongly binds its P5a region. Future ChIP‐sequence analysis will investigate whether other genes share the *Cacna1s* consensus sequence for TnT3 binding.

We also report here that the TnT3 endogenous cleavage site in muscle *in vivo* is compatible with calpain activity, which may be subtle and recurrent rather than massive because calpain inhibition significantly modified TnT3 fragmentation but not overall levels of TnT3 or major motor proteins as our analysis of myosin isoforms, actin, and titin shows. Thus, increased muscle susceptibility to trauma with aging (Faulkner *et al*., 1993) may involve calpain activation. Although TnCT contains a leucine zipper domain and therefore retains its capacity to bind DNA, it is likely to be less effective transcriptionally than TnFL due to its altered conformation (Wei & Jin, 2011), a key factor in protein/DNA interactions.

Our studies indicate that TnT3‐mediated *Cacna1* transcription is a primary but not the only mechanism regulation Cav1.1 expression (Zheng *et al*., 2002b). Restoring nuclear levels of FL‐TnT3 by calpain inhibition significantly recovers Cav1.1 expression but we do not know how completely. Recently, we demonstrated that increased Cavβ1a expression contributes to decreased Cav1.1 expression and muscle weakness with aging; however, the underlying mechanism is not gene transcription, because in young mice, Cav1.1 mRNA does not decline with Cavβ1a overexpression (Taylor *et al*., 2009). We also demonstrated that JP45 regulates Cav1.1 expression through a mechanism independent of gene transcription (Delbono *et al*., 2007, 2012). Aging skeletal muscle undergoes chronic denervation, but whether that accounts for the accumulation of nuclear TnCT remains to be examined.

Endogenous skeletal muscle calpain activity is inhibited by systemic administration of BDA‐410 in aging sedentary mice. BDA‐410 is a synthetic Leu‐Leu peptidomimetic that significantly attenuates various disease conditions (Carragher, 2006), including memory and synaptic transmission in a mouse model of Alzheimer disease and signs of premature aging in a mouse model of *kotho* deficiency (Manya *et al*., 2002; Trinchese *et al*., 2008). BDA‐410 strongly and reversibly inhibits cysteine proteases but not serine or aspartic proteinases. Its cyclopropenone group attracts the hydrogen of the –SH residues of cysteines contained in the calpain molecule (Nabeshima *et al*., 2014). BDA‐410‐induced calpain inhibition stabilizes nuclear TnT3 integrity and Cav1.1 protein levels in skeletal muscle *in vivo* and enhances muscle force, but not fatigue, in old mice, which is at least partially explained by its lack of effect on fiber type.

TnT3 is a master regulator of the two major calcium systems that mediate muscle mechanical output, contractile protein function and ECC. Publications from our laboratory and others indicate that weakness in old age is the result of decreased muscle mass and muscle‐specific force (force/CSA) (Delbono, 2003, 2011). BDA‐410 increases force development in soleus muscle, which is a ~ 50/50 mixture of fast and slow fibers, and because TnT3 is only expressed in fast fibers (type‐II), we predict a larger effect on pure fast‐twitch muscles, such as EDL and tibialis anterior. The improved function is associated with enhanced contraction efficiency but no change in muscle CSA. Hence, impaired intrinsic force‐generating capacity with age may be partially due to alterations in ECC (Tang *et al*., 2011). We (Renganathan *et al*., 1997) and others (Ryan *et al*., 2000) reported diminished Cav1.1 expression with aging, which increases uncoupled RyR1s and decreases sarcoplasmic reticulum Ca^2+^ release, leading to excitation–contraction uncoupling. Here, we postulate that increased TnCT and decreased TnFL in the nucleus impair *Cacna1s* transcription, reducing muscle force in aging sedentary mice. Our results also support the potential benefits of a short trial to determine whether calpain inhibition increases muscle force in older sedentary humans.

## Experimental procedures

### Mice

Old (23–25 months) female C57BL/6 mice were obtained from the National Institute on Aging (NIA), and housed in the pathogen‐free Animal Research Program of WFSM at 20–23 °C and 12:12‐h dark–light cycle. The mice were fed *ad libitum* and had continuous access to drinking water. Mice were sacrificed by decapitation with a guillotine and thoroughly inspected for gross pathology. Animal handling and procedures followed a protocol approved by the WFSM Animal Care and Use Committee.

### Cell culture and transfection

The mouse muscle cell line C2C12 was cultured as described (Zhang *et al*., 2013a,b). Briefly, C2C12 myoblasts were plated on tissue culture dishes or glass coverslips coated with 0.5% gelatin in growth medium (GM) consisting of Dulbecco's modified Eagle medium (DMEM, 1 g L^−1^ glucose) containing 10% FBS (Atlanta Biologicals, Atlanta, GA, USA) and 2 mm Glutamax (Invitrogen, Carlsbad, CA, USA). To induce differentiation and myotube formation, cells were switched to low serum differentiation medium (DM, 2% horse serum) and cultured for 3–5 days. Lipofactamine 2000 (Invitrogen) was used for cell transfection.

### RNA extraction, reverse transcription, and qPCR

Trizol reagent (Invitrogen) was used to extract total RNA from young and old mouse FDB muscles. Gene expression was analyzed by qPCR using Mx3000 (Stratagene, La Jolla, CA, USA). To determine TnT3 and GAPDH tissue expression, 10 ng of the total RNA was added to the PCR reaction tube with qRT–PCR master mix, Taqman primer/probes (Applied Biosystems, Foster City, CA, USA), superscript III reverse transcriptase (Invitrogen), and RNAse inhibitor (Promega, Fitchburg, WI, USA). The total reaction volume was 25 μL. The PCR parameters were 48 °C, 45 min × 1 cycle; 95 °C, 10 min × 1 cycle; 95 °C, 15 s × 1 cycle; and 60 °C, 1 min × 40 cycles.

### Dual luciferase assay

To measure *Cacna1s* promoter activity, a dual luciferase assay was carried out as described (Zheng *et al*., 2002a). Briefly, C2C12 cells were plated in GM on 35‐mm dishes (2 × 10^4^ cells per dish) until reaching 70% confluence. For cell transfection, a mixture of 2 μg of *Cacna1s* D2 promoter (Zheng *et al*., 2002a), 2 μg of either control shRNA (shC) or TnT3‐targeting shRNA (shT) plasmid, and 200 ng of the control vector pRL‐TK (Promega) was exposed to Lipofectamine 2000 following the manufacturer's protocol. Cells were cultured in DM for 5 days to induce differentiation before whole cell lysis. To perform the dual luciferase assay in muscle *in vivo*, 10 μg of *Cacna1s* D2 promoter, 0.6 μg of control vector pRL‐TK, and 10 μg of DsRed constructs (DsRed, TnFL/DsRed, or TnFL‐dNLS/DsRed) were mixed and electroporated into the FDB muscle as described (Zhang *et al*., 2013b). Three weeks later, FDB muscles were dissected, and cell lysis was induced using a passive lysis buffer (Promega). Luciferase and renilla activity were measured using a luminometer (Turner 20E, Sunnyvale, CA, USA). Values for the luciferase assay were normalized to renilla luciferase activity to minimize differences in the transfection efficiency of each experiment.

### TnT3 purification from mouse skeletal muscle

TnT was purified using following previous methods (Eisenberg & Kielley, 1974) with some modifications. After dissection, the homogenized TA muscle was washed 5–6 times with 50 mm KCl, 5 mm Tris pH 8, 1% Triton X‐100, 0.5 mm, phenylmethylsulfonyl fluoride, and 1 protease inhibitor tablet (SigmaFAST^™^; Sigma‐Aldrich, St. Louis, MO, USA) per 25 mL of buffer. The residue was further washed three times with ethanol and three times with acetone. The powder was allowed to dry in a fume hood and then stored at −20 °C until needed. The residue was extracted for 8 h at 5 °C with 15 mL g^−1^ residue of 1 m KCl, 25 mm Tris pH 8, 0.1 mm CaCl_2_, 0.1 mm dithiothreitol, and one protease inhibitor tablet per 25 mL. Following centrifugation, the supernatant was loaded onto a 1‐mL hydroxylapatite column equilibrated in 1M KCl and 10 mm sodium phosphate (pH 6.8). The column was washed with 3 volumes of the start buffer and troponin was eluted with 2 volumes of buffer with 66 mm Pi. Eluted samples were kept at −80 °C for later use. Eluted protein (3 μg) was used in each reaction subjected to EMSA.

### N‐terminal protein coupling with TMPP followed by 1D SDS‐PAGE separation and mass spectrometry

Nuclear protein (NP) extractions (100 μg) were precipitated with methanol/chloroform. Precipitates were resuspended in 200 μL labeling buffer (50 mm Tris‐HCl, 8 m urea, 2 m thiourea, pH 8.2, 1 mm phenylmethylsulfonyl fluoride, 1 mm EDTA, 5 mm TBP, protease inhibitor mixture, 10% CH_3_CN, 1% SDS). The proteins were labeled by adding 1.6 μL of 0.1 m of TMPP‐Ac‐OSu in CH_3_CN:water (2:8, v/v). After a quick mix, the reaction was maintained at room temperature for 1 h. Residual derivatizing reagent was quenched by adding 5 μL of 0.1 m hydroxylamine, quick mixed and incubated at room temperature for 1 h. Finally, 22.8 μL glycerol was added to reach a concentration of 10%. Each mixture of proteins (TMPP‐derivatized and nonlabeled NPs) was separated on a 12% 1D SDS‐PAGE. The gel was stained with Coomassie blue. Bands that are matched to the TnCT region (predetermined by immunoblot) were excised, processed with tryptic digestion and then analyzed by mass spectrometry using a Thermo LTQ Orbitrap Velos Pro high‐resolution mass spectrometer interfaced with a splitless multidimensional nanoLC system (Thermo Fisher, Waltham, MA, USA/Dionex, Sunnyvale, CA, USA) (Suh *et al*., 2010). Raw data files were searched against the mouse proteome using the Mascot search engine and proteome discoverer v. 1.4 (Thermo Fisher).

### Electrophoretic mobility shift assay

To verify direct interaction between TnT3 protein and the *Cacna1s* promoter region, Electrophoretic mobility shift assay (EMSA) was performed according to the protocol described in the Odyssey Infrared EMSA kit. Briefly, 3 μg of purified TnT3 was incubated with synthesized oligo nucleic acids labeled with fluorescent IRdye700 (LI‐COR Biosciences, Lincoln, NE, USA). Oligo nucleic acids of the same sequence but without a dye label were used at a concentration 200‐fold higher in competition experiments, and TnT3 antibody was used in a supershift assay. The oligos (Table S1) were selected based on our reported *Cacna1s* promoter sequence (Zheng *et al*., 2004) and our chromatin immunoprecipitation data. After the binding reaction, the mixture was loaded onto a Mini‐PROTEAN TBE precast gel (5%; Bio‐Rad, Hercules, CA), run in 0.5% TBE at 70V for 1 h, and then imaged directly with an Odyssey Infrared Imager (LI‐COR Biosciences).

### Microscopy and image analysis

C2C12 cells cultured on coverslips, fixed with 4% paraformaldehyde for 15 min, and their membranes permeabilized for 5 min with 0.5% Triton X‐100 in PBS buffer at RT. After three washes with PBS, cells were incubated for 1 h in blocking buffer (PBS with 10% normal goat serum [Sigma, St. Louis, MO, USA]) and labeled with primary and secondary antibodies for 2 and 1 h, respectively. Cells were counterstained with Hoechst 33342 (Invitrogen) and mounted in DAKO fluorescent mounting medium (Carpinteria, CA, USA).

Samples were imaged on an inverted motorized fluorescent microscope (Olympus, IX81, Tokyo, Japan) with an Orca‐R2 Hamamatsu CCD camera (Hamamatsu, Japan). The camera driver and image acquisition were controlled with a MetaMorph Imaging System (Olympus). Digital image files representing three independent experiments were transferred to Photoshop 7.0/CS5 to assemble montages.

### Reagents and antibodies

Control nontargeting shRNA (shC) and TnT3‐targeting shRNA (shT) were purchased from Sigma. Rabbit anti‐TnT3 polyclonal antibody was obtained from Aviva Systems Biology (San Diego, CA, USA); mouse anti‐TnT1 CT3 antibody and mouse anti‐Cavβ1a from Developmental Studies Hybridoma Bank (DSHB, Iowa City, IA, USA); and mouse monoclonal IIF7 to Cav1.1 was a generous gift from Dr. Kevin P. Campbell (University of Iowa, IA, USA). Mouse antimyosin heavy chain (MHC) MF20 was purchased from DSHB, and mouse anti‐actin from Millipore (Billerica, MA, USA). Alexa 488‐conjugated anti‐mouse and anti‐rabbit IgG were purchased from Invitrogen. NA931V goat anti‐mouse and NA9340V donkey anti‐rabbit (Amersham Health, Marlborough, MA, USA) were used as secondary antibodies for immunoblotting.

#### BDA‐410 administration

Mice were treated daily for 21 days with either 1% Tween 80 in normal saline (vehicle) or calpain inhibitor BDA‐410 [(2S)‐N‐(1S)‐1‐[(S)‐Hydroxy(3‐oxo‐2‐phenyl‐1‐cyclopropen‐1‐yl)methyl]‐2‐methylpropyl‐2‐benzenesulfonylamino‐4‐methylpentanamide], a cyclopropenone derivative synthetized by Mitsubishi Tanabe Pharma Corporation (Yokohama, Japan) dissolved in vehicle at 30 mg kg^−1^ day^−1^, and received 10 mL kg^−1^ by oral gavage with a straight 20G feeding needle (Fine Sciences Tools, Foster City, CA, USA).

### *In vivo* muscle function recording

Force was recorded in the posterior compartment of the right hindlimb (gastrocnemius, soleus, and plantaris muscles) using an *in vivo* force transducer (Aurora Scientific, Aurora, ON, Canada) attached to the mouse footplate. Mice were anesthetized with 2% inhaled isofluorane at a constant flow rate of 2 L min^−1^. After removal of the animal's fur, the common peroneal nerve was located and an electrode was placed over the nerve. Resting tension, muscle length, and stimulation current were iteratively adjusted for each muscle to obtain optimal twitch force. The nerve was then stimulated to obtain force–frequency curves as described (Files *et al*., 2012).

#### *In vivo* fatigue recording

*In vivo* fatigue was recorded before and after treatment using a forced Exer 6 lane treadmill apparatus (Columbus Instruments, Columbus, OH, USA), as described (Delbono *et al*., 2012). On consecutive days, mice were allowed to run on the treadmill for 5 min at 10 m min^−1^ on days 1 and 2 and 5 min at 10 m min^−1^ followed by 2 m min^−1^ increments until reaching 20 m min^−1^ for 2 min each period on day 3. The treadmill was not inclined. After training, mouse performance was recorded. The initial speed was 10 m min^−1^ to a maximum of 5 min, and then, we increased the pace by 2 m min^−1^ every 2 min to a maximum of 24 m min^−1^ or until exhaustion. We considered the mouse exhausted when it remained at least 10 s in the electric shock area of the treadmill. Maximal tolerated speed was recorded.

### Inverted‐cling grip test

Grip strength was assessed using Kondziela's inverted screen test before and after mouse treatment. This procedure provides a measure of overall strength and muscular endurance of the mouse. The mice were allowed to acclimate in the experimental room for 10 min before testing, and the time they held from the net was computed. The results were the average of three consecutive trials.

### *Ex vivo* whole muscle contraction

Soleus muscles were dissected, placed in cold Krebs's solution, and most of the surrounding connective tissue was removed. Aluminum T‐shaped clips were used to attach the muscle to a 407A force transducer (Aurora Scientific; compliance: 0.01 μm mN^−1^, resonant frequency: 4.0 kHz) and a length controller (González *et al*., 2003). The muscle was continually perfused with buffered recording solution (in mm: NaCl 121, KCl 5, CaCl_2_ 1.8, MgCl_2_ 0.5, NaH_2_PO_4_ 0.4, NaHCO_3_ 24.0, glucose 5.5, *d*‐tubocurarine chloride hydrate 0.015) bubbled with a mixture of 5% CO_2_ and 95% O_2_ to maintain pH 7.4. The muscle was allowed to balance for 15 min before direct stimulation by an electrical field generated between two parallel platinum electrodes connected to a stimulator. The muscle length was adjusted until a single stimulus pulse or a train of pulses elicited maximum force during twitch or tetanus (optimal length [Lo]) under isometric conditions. After attaining Lo, the muscle was allowed to rest for 8 min, and subsequently, the force–frequency relationship was recorded at 1, 5, 10, 20, 50, 100, and 150 Hz using 400‐ms trains of pulses. The interval between trains of pulses was set according to the duration of the stimulus (0.5–8 min). Muscle fatigue was recorded in response to 400 ms/150 Hz supramaximal pulses applied for a maximum of 5 min. Experiments were performed at 22 °C. Data were analyzed using asi600a Digital Controller software (Aurora).

### Skeletal muscle dissection and preparation for protein, RNA analysis or histology

After *in vivo* functional recordings, muscles were collected in RNase‐free conditions. Muscles assigned to protein and RNA analysis were snap‐frozen in liquid nitrogen and stored at −80 °C. Muscles for histological evaluations were fixed in 4% paraformaldehyde at resting length, cryopreserved by sucrose gradient, embedded in O.C.T compound (Tissue‐Tek^®^, Sakura Finetek, Torrance, CA, USA), frozen in dry ice‐chilled isopentane, and stored at −80 °C.

#### Muscle cross‐sectional area

The muscle cross‐sectional area (CSA) was calculated by dividing wet mass by the product of the fiber length and 1.06 g cm^−3^, the density of mammalian skeletal muscle (Brooks & Faulkner, 1988). The fiber length was estimated by multiplying the muscle length at Lo by 0.71, the ratio between fiber length and muscle length for mouse soleus muscle (Brooks & Faulkner, 1988). The value of the CSA was later used to calculate muscle‐specific force (Newton cm^−2^).

##### SDS‐polyacrylamide gel electrophoresis (PAGE) and Western blotting

Methods for FL‐TnT and fragments in cellular subcompartments followed described procedures (Zhang *et al*., 2013b). The frozen soleus muscles or EDL muscle were cut into small pieces in a tube on dry ice. SDS‐PAGE sample buffer containing 2% SDS and 3% *β*‐mercaptoethanol, pH 8.8, was added to the tube at 40‐fold of the muscle weight (μL mg^−1^) for homogenizing the muscle tissue using a high‐speed mechanical homogenizer. The SDS‐PAGE samples were then heated at 80 °C for 5 min and centrifuged at 14 000 *g* in a microcentrifuge for 5 min to remove insoluble debris. The supernatant was resolved on SDS‐gels with 14% acrylamide, bisacrylamide at a ratio of 180:1 prepared in a modified Laemmli buffer system, in which both stacking and resolving gels were at pH 8.8 or 2 ~ 12% gradient gel with acrylamide, bisacrylamide ratio of 180:1. The resolved protein bands were visualized by Coomassie Blue R‐250 staining. The actin bands were quantified using ImageJ software (National Institutes of Health, NIH, Bethesda, MD, USA) to normalize sample loading.

Duplicate SDS‐gels were transferred to nitrocellulose membrane using a Bio‐Rad semidry electrical transfer device at constant current of 5 mA cm^−2^ for 15 min. The blotted membranes were blocked with 1% BSA in Tris‐buffered saline (TBS, 150 mm NaCl, 50 mm Tris‐HCl, pH 7.5) at room temperature with shaking for 30 min. The blocked membrane was probed at 4 °C overnight with anti‐TnI monoclonal antibody (mAb) TnI‐1 (Jin *et al*., 2001), antislow TnT mAb CT3 or antifast TnT mAb T12 (Jin & Chong, 2010) diluted in TBS containing 0.1% BSA. The membranes were then washed three times for 7 min each with TBS containing 0.5% Triton X‐100 and 0.05% SDS and three times for 3 min each with TBS before incubation with alkaline phosphatase‐labeled goat anti‐mouse IgG second antibody (Santa Cruz Biotechnology, Dallas, TX, USA) at room temperature for 1 h. The membranes were washed again and developed in 5‐bromo‐4‐chloro‐3‐indolyl phosphate/nitro blue tetrazolium substrate solution to visualize the protein bands recognized by the specific mAbs.

Myosin heavy chain (MHC) isoforms were examined as described previously (Feng *et al*., 2009). SDS‐gel sample equal to 5 μg of muscle tissue was resolved on SDS‐polyacrylamide gel with 8% acrylamide, bisacrylamide at the ratio of 50:1 containing 30% glycerol in an icebox for 24 h. The gels were stained with Coomassie Blue R250, and the relative amounts of MHC isoforms were quantified by two‐dimensional densitometry using ImageJ.

#### Calpain activity

After completion of the 3‐week treatment mice were sacrificed, hamstring muscles dissected and snap‐frozen in liquid nitrogen. For calpain activity assay, we followed described methods (Supinski *et al*., 2009). Briefly, 100 μg protein was added to a buffer containing: 50 mm HEPES, 100 mm NaCl, 0.1% NaCl, 0.1% CHAPS, 10 mm DTT, 1 mm EDTA, 10% glycerol, pH: 7.4, and a fluorogenic substrate cleaved by calpain [succinyl‐Leu‐Leu‐Val‐Tyr‐7‐amido‐4‐methyl‐coumarin (Suc‐LLVY‐AMC)]. Duplicate determinations were made for each sample using a mixture of gastrocnemius muscle homogenate, assay buffer, fluorogenic substrate, and highly specific calpain inhibitor (0.1 mg mL^−1^ calpain inhibitor III, carbobenzoxy‐valinyl‐phenylalaninal). Immediately after the addition of the fluorogenic substrate, a baseline fluorescent measurement of AMC (7‐amino‐4‐methylcoumarin) was performed using a spectrofluorophotometer (excitation frequency of 360 nm and an emission frequency of 460 nm, Molecular Devices, Sunnyvale, CA, USA). AMC standards were used to create a calibration curve, and activity was quantified as nanomoles of AMC generated per minute per milligram of tissue homogenate protein. The difference between AMC generation from incubation of homogenates with Suc‐LLVY‐AMC in the presence and absence of calpain inhibitor III was taken as an index of calpain activity; note that calpain inhibitor III blocks calpain activity but does not inhibit the proteasome or other chymotrypsin‐like proteases (Supinski *et al*., 2009).

### Statistical analysis

Data were analyzed using sigmaplot 12.5 (Systat Software, San José, CA, USA) or prism 5.0a (GraphPad Software, Inc., La Jolla, CA, USA). All data are presented as means ± SEM and reported *P* values are the result of one‐sided tests. The alpha level was set at *P* < 0.05. Student's *t*‐test or analysis of variance (ANOVA) was used to compare experimental groups where appropriate.

## Author contributions

T.Z. designed and performed experiments, analyzed the data, and wrote the manuscript. A.S.P, J.R, C.F., J.‐P.J, and J.C. performed experiments, analyzed the data, and contributed to manuscript writing. A.B., M.L.M., and ZMW. performed the experiments. H.F., D.C.F., X.F., and L.P. performed and analyzed the experiments. O.D. designed the experiments and wrote the manuscript.

## Funding

This work was supported by the National Institutes of Health grants, AG13934 and AG15820 to Osvaldo Delbono, AR048816 to J.‐P. Jin, the Wake Forest Claude D. Pepper Older Americans Independence Center P30‐AG21332, and by a Glenn/AFAR Scholarship for Research in the Biology of Aging to Alexander Birbrair.

## Conflict of interest

The authors declare no competing financial interests.

## Supporting information


**Fig. S1** Sequence alignment of the *Cacna1s* promoter region that binds to TnT3 in ChIP assays.

**Fig. S2** Mapping of TnT3 endogenous cleavage site in muscle *in vivo*.

**Fig. S3** BDA‐410 inhibits calpain activity in mouse skeletal muscle.

**Fig. S4** Effects of BDA‐410 on muscle fatigue and endurance.

**Fig. S5** Calpain inhibition does not modify the myosin/actin ratio in fast and slow muscles from old mice.

**Fig. S6** Calpain inhibition does not modify fast or slow troponin T or troponin I.

**Table S1** EMSA oligonucleotide sequences.
