# Supplementary figures and images for: Calpain inhibition rescues troponin T3 fragmentation, increases Cav1.1, and enhances skeletal muscle force in aging sedentary mice
Source: Aging Cell. 2016 Feb 19;15(3):488–98. doi: 10.1111/acel.12453 (PMC4854922; doi:10.1111/acel.12453)

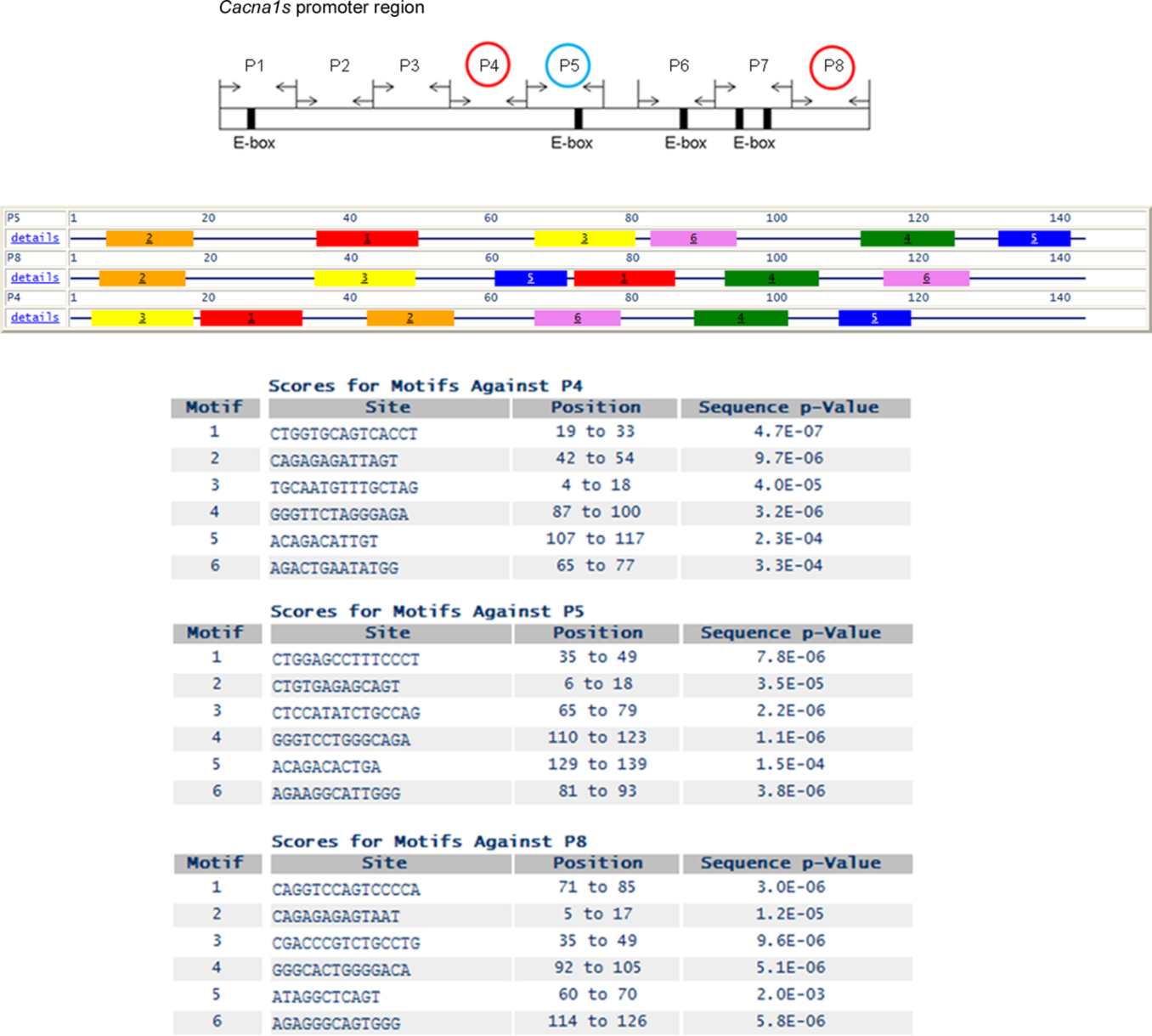

Supplement: Supplementary file 1 — Fig. S1 Sequence alignment of the Cacna1s promoter region that binds to TnT3 in ChIP assays. [file ACEL-15-488-s001.tif]

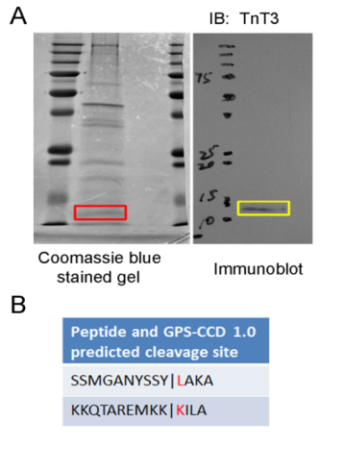

Supplement: Supplementary file 2 — Fig. S2 Mapping of TnT3 endogenous cleavage site in muscle in vivo. [file ACEL-15-488-s002.tif]

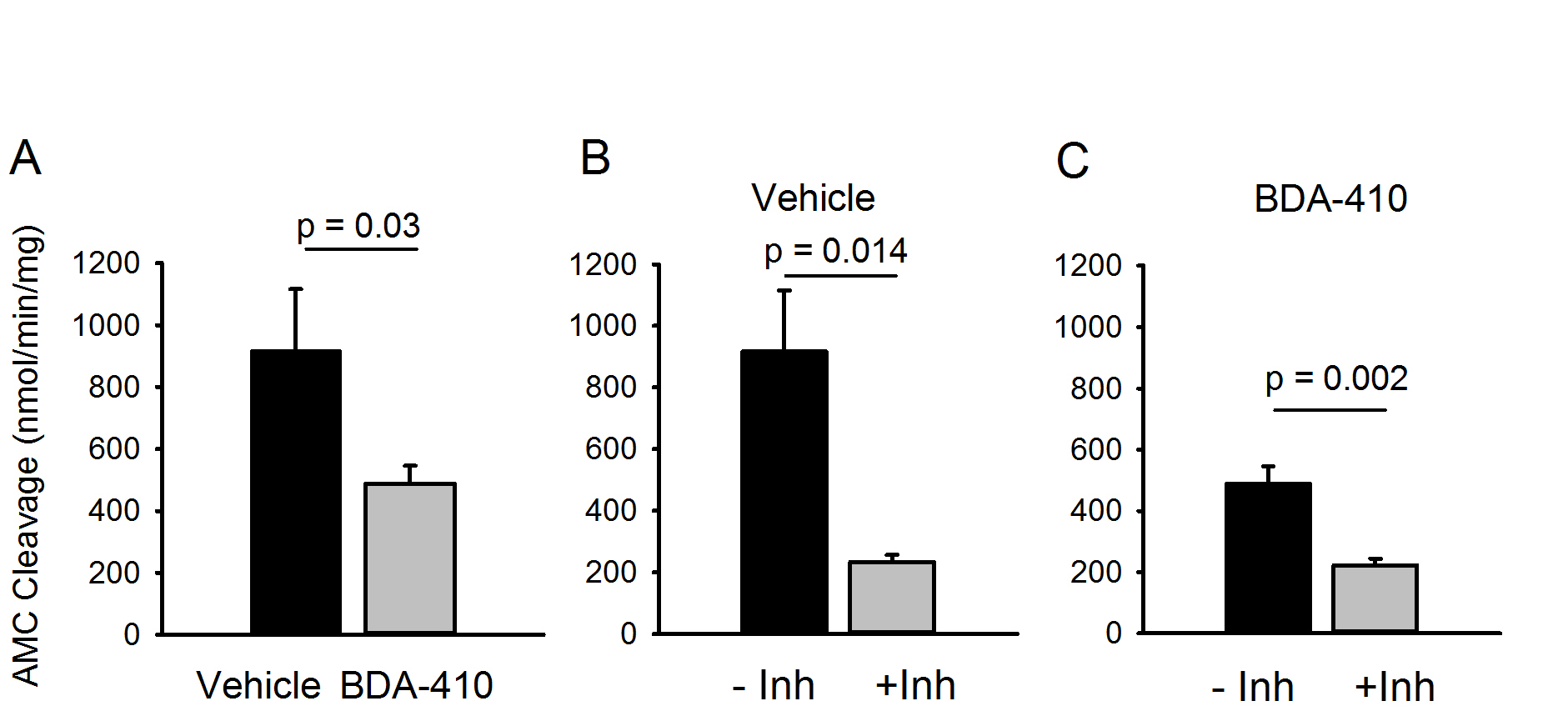

Supplement: Supplementary file 3 — Fig. S3 BDA‐410 inhibits calpain activity in mouse skeletal muscle. [file ACEL-15-488-s003.jpg]

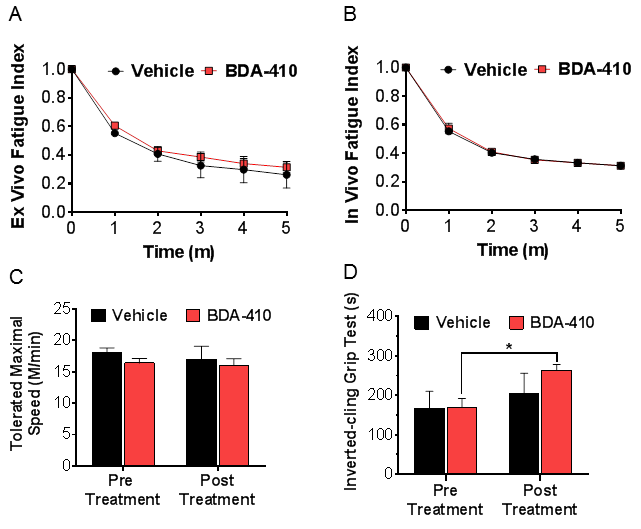

Supplement: Supplementary file 4 — Fig. S4 Effects of BDA‐410 on muscle fatigue and endurance. [file ACEL-15-488-s004.tif]

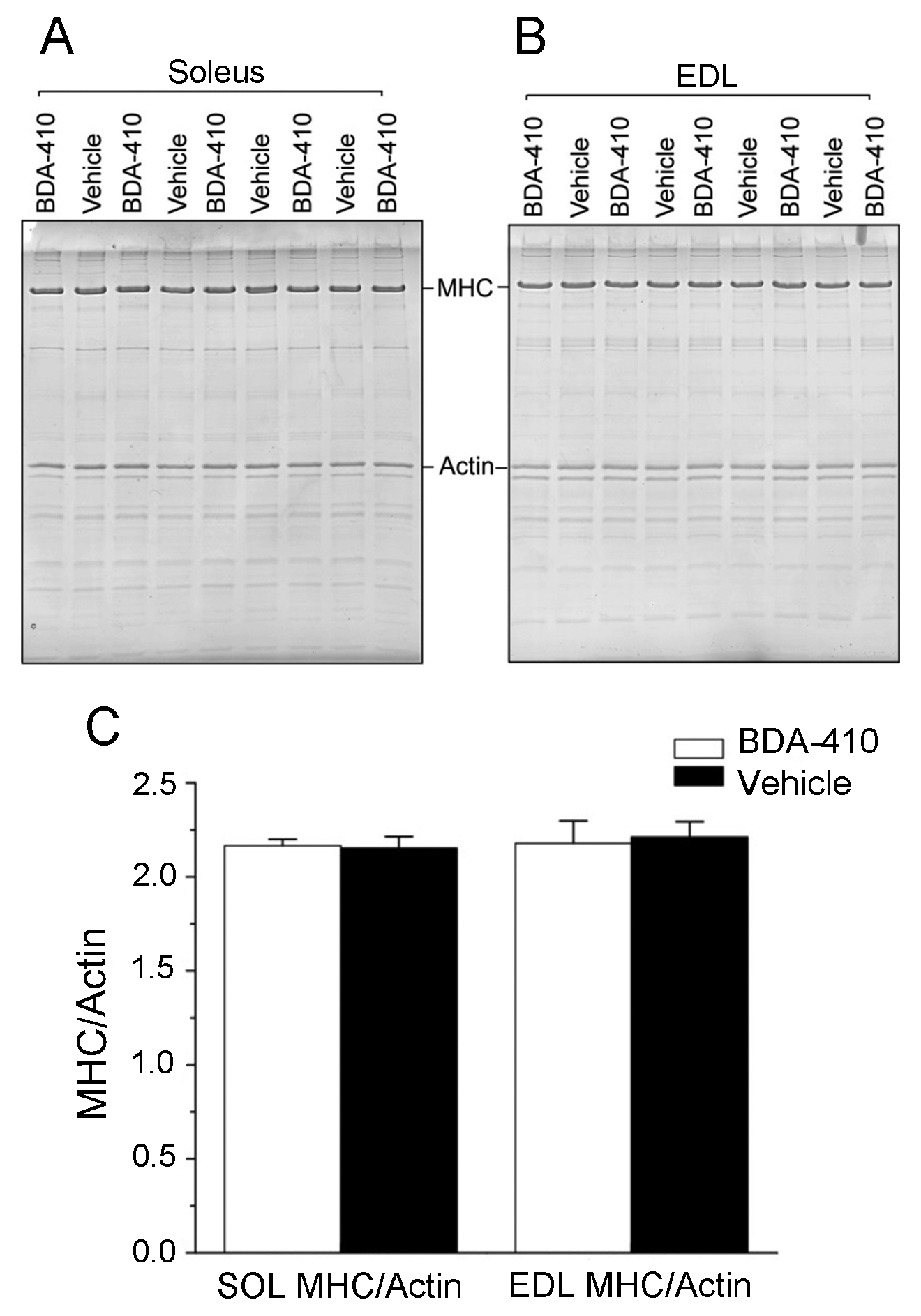

Supplement: Supplementary file 5 — Fig. S5 Calpain inhibition does not modify the myosin/actin ratio in fast and slow muscles from old mice. [file ACEL-15-488-s005.tif]

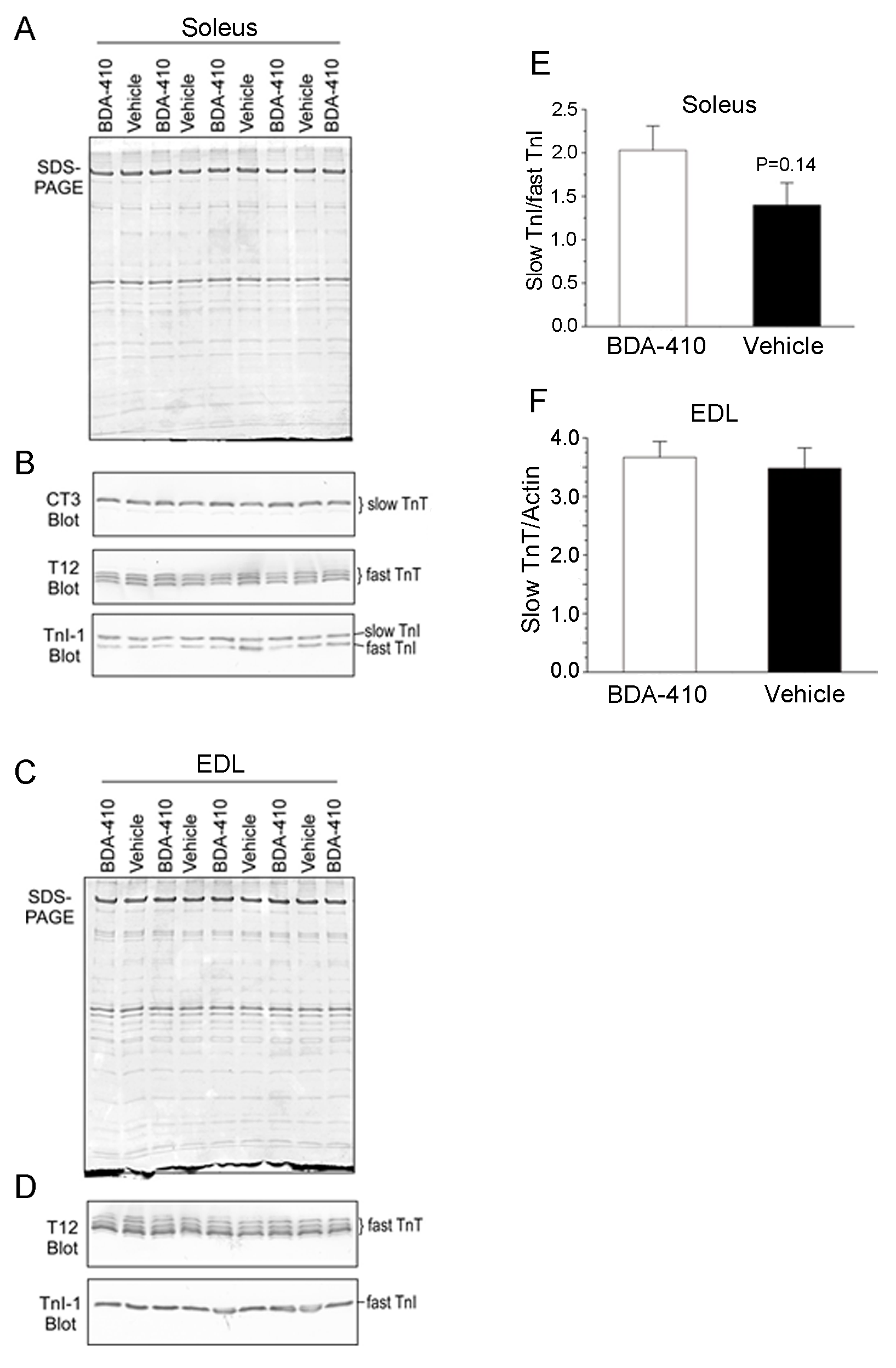

Supplement: Supplementary file 6 — Fig. S6 Calpain inhibition does not modify fast or slow troponin T or troponin I. [file ACEL-15-488-s006.tif]
