# Supplementary material for: Calpain inhibition rescues troponin T3 fragmentation, increases Cav1.1, and enhances skeletal muscle force in aging sedentary mice
Source: Aging Cell. 2016 Feb 19;15(3):488–98. doi: 10.1111/acel.12453 (PMC4854922; doi:10.1111/acel.12453)
Supplement: Supplementary file 7 — Table S1 EMSA oligonucleotide sequences. [file ACEL-15-488-s007.docx]

**Table S1. EMSA oligonucleotide sequences**

| **Names** | **Sequences (sense oligo)** | **Figs** |
| --- | --- | --- |
| **P5** | ATCTGCCAGAAGAAGGCATTGGGGGCGG**C**AA**GTG**TGAGAGGGTCCTGGGCAGATCCTGACAGACACTGAG | **2B, F** |
| **P5a(-wt)** | ATCTGCCAGAAGAAGGCATTGGGGGCGG | **2F(H)** |
| **P5b** | TGAGAGGGTCCTGGGCAGATCCTGACAGACACTGAG | **2F** |
| **P5c** | ATCTGCCAGAAGAAGGCATTGGGGGCGG**A**AA**AAA**TGAGAGGGTCCTGGGCAGATCCTGACAGACACTGAG | **2F** |
| **P5a-R3m** | A**AAAAAA**A**A**AA**G**AA**GGC**A**TTGGG**GGCGG | **2H** |
| **P5a-R6m** | A**TCTGCC**A**G**AA**A**AA**AAA**A**AAAAA**GGCGG | **2H** |
| **Control 1: MEME- predicted Cavβ1a binding motif** | TGGTGGCGCATGCCTTTAATCCCAGCACTCGGGAG | **2B** |
| **Control 2: Myogenin promoter region** | CGTCTTGATGTGCAGCAACAGCTTAGA | **2B** |

Highlighted sequences match E-box or consensus motifs (see Fig. 2D).
